# Optimized microbial DNA extraction from diarrheic stools

**DOI:** 10.1186/1756-0500-5-702

**Published:** 2012-12-28

**Authors:** Emilie Donatin, Michel Drancourt

**Affiliations:** 1 Aix Marseille Université, URMITE, UMR63 CNRS 7278, IRD 198, Inserm 1095, 13005, Marseille, France; 2 Unité des Rickettsies, Faculté de Médecine, 27, Boulevard Jean Moulin, Marseille-Cedex 5, France

**Keywords:** DNA extraction, Lyophilization, Diarrheal stools

## Abstract

**Background:**

The detection of enteropathogens in stool specimens increasingly relies on the detection of specific nucleic acid sequences. We observed that such detection was hampered in diarrheic stool specimens and we set-up an improved protocol combining lyophilization of stools prior to a semi-automated DNA extraction.

**Findings:**

A total of 41 human diarrheic stool specimens comprising of 35 specimens negative for enteropathogens and six specimens positive for *Salmonella enterica* in culture, were prospectively studied. One 1-mL aliquot of each specimen was lyophilised and total DNA was extracted from lyophilised and non-lyophilised aliquots by combining automatic and phenol-chloroform DNA extraction. DNA was incorporated into real-time PCRs targeting the 16S rRNA gene of Bacteria and the archaea *Methanobrevibacter smithii* and the chorismate synthase gene of *S. enterica*. Whereas negative controls consisting in DNA-free water remained negative, *M. smithii* was detected in 26/41 (63.4%) non-lyophilised (Ct value 28.78 ± 9.1) versus 39/41 (95.1%) lyophilised aliquots (Ct value 22.04 ± 5.5); bacterial 16S rRNA was detected in 33/41 (80.5%) non-lyophilised (Ct value 28.11 ± 5.9) versus 40/41 (97.6%) lyophilised aliquots (Ct value 24.94 ± 6.6); and *S. enterica* was detected in 6/6 (100%) non-lyophilized and lyophilized aliquots (Ct value 26.98 ± 4.55 and 26.16 ± 4.97, respectively). *S. enterica* was not detected in the 35 remaining diarrheal-stool specimens. The proportion of positive specimens was significantly higher after lyophilization for the detection of *M. smithii* (p = 0.00043) and Bacteria (p = 0.015).

**Conclusion:**

Lyophilization of diarrheic stool specimens significantly increases the PCR-based detection of microorganisms. The semi-automated protocol described here could be routinely used for the molecular diagnosis of infectious diarrhea.

## Findings

Infectious diarrhea is a leading cause of mortality and morbidity worldwide, being responsible for 2.16 million deaths a year, including 1.5 million pediatric deaths (3.7% of deaths in the world) ( http://who.int/en/). Infectious diarrhea is caused by a wide spectrum of enteropathogens including the bacteria *Salmonella* spp., enteropathogenic *Escherichia coli*, *Shigella* spp.*, Yersinia* spp.*, Campylobacter* spp. and *Clostridium difficile*[1] and noroviruses, rotaviruses, toroviruses, coronaviruses, astroviruses, enteroviruses and adenoviruses, all pathogens reportedly causing 50% of cases of diarrhea [2]. As most of these pathogens are fastidious to culture, the direct diagnosis of infectious diarrhea relies on the detection of enteropathogen-specific antigen by immunochromatographic assays [3-6] and the detection of enteropathogen-specific nucleic sequences by PCR, real-time PCR and DNA microarray [7,8]. Later detection however is hampered by the presence of PCR inhibitors [9] and the dilution of targeted pathogen in watery stools. When the normal excretion of water in stool varies between 150 and 200 mL every 24 hours [10], water excretion may increase up to one liter in diarrheic stools [11]. Previous studies showed that a preliminary enrichment step applied to stool increases the detection of enteropathogen DNA [12,13]. However, such an enrichment step delays molecular testing for up to 48 hours. We therefore aimed to optimize the DNA extraction protocol to target both bacteria and archaea in diarrheal stool specimens.

A total of 56 stool specimens (15 non-diarrheal specimens and 41 diarrheal stool) were prospectively collected in 56 individuals as part of the routine diagnostic activity in the Microbiology laboratory, Timone Hospital, Méditerranée Infection, Marseille, France. A total of 50 stools were negative for the routine detection of pathogenic bacteria and six diarrheal stool specimens yielded *Salmonella enterica* in culture. No written consent was needed for this work in accordance with the “Loi n° 2004–800 relative à la bioéthique” published in the “Journal Officiel de la République Française” the 6 August 2004 since no additional sample was taken for the study. According to this law, patients were informed that stool specimens could be used for anonymised studies. This study was approved by the local ethic committee of the Institut Fédératif de Recherche 48, Faculty of Medicine, Marseille, France under the reference number 08–002. Non-diarrheal and diarrheal stool specimens were treated separately.

In a first step, the 15 non-diarrheal stool specimens were diluted 1:5 in sterile phosphate buffer (PBS) in order to mimic diarrheal stool specimens. These 15 diluted specimens were divided into two aliquots. One aliquot was frozen for 4 hours at −20°C and freeze-dried lyophilized for 24 hours in 1-mL glass containers (Dominique Dustcher, Brumath, France) using a LYOVAC GTZ instrument (Leybold Hereaeus, Roubaix, France). After lyophilization, stool specimens were regenerated into 250 μL PBS resulting in a four-fold concentration of the stool specimens. The second aliquot was not lyophilized. The DNA extraction was then performed for the two aliquots using a semi-automated protocol combining the EZ1 Advanced XL extractor (Qiagen, Courtaboeuf, France) and a phenol-chloroform DNA extraction [14]. For the extraction protocol, a 250 μL-aliquot of the resuspension of lyophilized sample was transferred into a sterile screw-cap Eppendorf tube containing 0.3 g of acid-washed beads (≤106 mm; Sigma, Saint-Quentin-Fallavier, France) and shaken in a FastPrep BIO 101 apparatus (Qbiogene, Strasbourg, France) at level 6.5 (full speed) for 180 s to achieve mechanical lysis. The supernatant was collected and incubated overnight at 56°C with 180 mL of lysis buffer and 25 μL of proteinase K (20 mg/mL) from the Qiagen EZ1® DNA Tissue kit. A 100 μL-volume of total DNA was then extracted from 200 μL specimens using the Qiagen EZ1® DNA Tissue kit in the EZ1 Advanced XL extractor. A final step of phenol-chloroform extraction was performed. Negative controls consisting of sterile DNA-free water were introduced at all steps and underwent the same extraction process that was used for the stool specimens. We analyzed specimens by real-time PCR with systems targeting a 128-bp portion of the 16S rRNA gene of *M. smithii* (V_4_ region, positions 648–739, *M. smithii* Genbank accession number IQ 346750), Bacteria 16S rRNA gene (V_3_-V_4_ inter spacer region, positions 555–639, *Escherichia coli* Genbank accession number FN 821375) and a specific system targeting a 121-bp part of the chorismate synthase gene of *Salmonella enterica* (Table 1). These real-time PCR systems were designed in our laboratory using the Primer3 software ( http://frodo.wi.mit.edu/). The specificity of primers and probes (using a pre-test 100% coverage and 100% identity for the targeted pathogens criteria) was tested using megaBlast against the nr-NCBI database ( http://blast.ncbi.nlm.nih.gov/Blast.cgi). In-silico analyses indicated that the “universal” bacterial system recognized more than 18,000 bacterial species, but also 300 archeal species and less than 10 eukaryota. Among the 18,000 bacterial species the “universal” system missed Chlamydiae and TM7 divisions, but allowed the detection of some Spirochetes and Synergistes. Whereas the *M. smithii* system forward primer system matches with other *Methanobrevibacter* species including *M. oralis*, *M. arboriphilus*, *M. ruminantum* or *M. wolinii*, reverser primer and the probe were found to be specific for *M. smithii*. The system targeting *S. enterica* was designed based on the complete genome of *S. enterica* subspecies *enterica* Typhimurium strain 798 (GenBank accession number CP003386). Primers and probe of this system were found to be specific for *S. enterica,* allowing the amplification of *S. enterica* serovars Enteritidis, Heidelberg, Typhimurium, Gallinarum, Paratyphi A and B, Saintpaul, Schwarzengrund, Dublin and Newport. Primers and probes were diluted to 20 pmol/μL and 25 pmol/μL respectively. PCR mixtures (20 μL) contained 10 μL Master Mix (Qiagen), 0.5 μL each primer and probe, 0.5 μL uracil-DNA-glycosylase (UDG) (Invitrogen-Life Technologies, Saint Aubin, France), four μL water and four μL DNA. Real-time PCRs incorporated two-min UDG decontamination at 50°C and ten-min denaturation at 95°C followed by 40 cycles (45 cycles for *M. smithii* detection) of one second at 95°C, 35 seconds at 60°C and 45 seconds at 72°C. Each specific real-time PCR incorporated a positive and a negative control. The cut-off of positivity was set-up at a 38 cycle threshold (Ct). All specimens were tested in duplicate.

**Table 1:** Real-time PCR systems used to test the efficiency of stool concentration by lyophilization

**Bacteria**	**Target**	**Sequence (5’ – 3’)**	**Length (bp)**
*Methanobrevibacter smithii*	16S rRNA	CCGGGTATCTAATCCGGTTC	20
		CTCCCAGGGTAGAGGTGAAA	20
		CCGTCAGAATCGTTCCAGTCAG	22
All bacteria	16S rRNA	AGAGTTTGATCMTGGCTCAG	20
		TTACCGCGGCKGCTGGCAC	19
		CCAKACTCCTACGGGAGGCAGCAG	24
*Salmonella enterica*	Chorismate synthase	CAAGAAATACCTGGCGGAAA	20
		CGGGACAAAAGAACGGATTA	20
		GTTCGGCATCGAAATCCGCG	20

M = C or A.

K = T, U or G.

*M. smithii* was detected in 11/15 (73.3%) non-lyophilized aliquots with a cycle threshold (Ct) value of 33.73 ± 7.5 versus 14/15 (93.3%) lyophilized aliquots (Ct value of 24.38 ± 7.3) (Table 2). The universal system gave positive results for 13/15 (86.7%) non-lyophilized aliquots (Ct value of 30.85 ± 5.9) versus 15/15 (100%) lyophilized aliquots (Ct value of 23.68 ± 5.9) (Table 3). All these 15 non-diarrheal specimens were negative for the specific detection of *S. enterica* before and after lyophilization. There is no significant difference (student test) before and after lyophilization for the detection of *M. smithii*, Bacteria and *S. enterica*.

**Table 2:** ***M. smithii *real-time PCR detection in 15 non-diarrheal stool specimens diluted in 1:5 in sterile phosphate buffer, before and after lyophilization (Cycle threshold [Ct] value)**

**Sample n°**	**1**	**2**	**3**	**4**	**5**	**6**	**7**	**8**	**9**	**10**	**11**	**12**	**13**	**14**	**15**
Without lyophilization	40.5	NA	38.93	36.43	26.38	NA	27.92	39.63	NA	22.27	33.43	43.61	38.53	NA	23.39
With lyophilization	34.39	34.74	33.93	16.97	16.84	11.72	20.16	30.24	19.76	23.11	22.65	NA	31.15	23.28	22.41

NA, not amplified.

**Table 3:** All bacteria real-time PCR detection with a universal system in 15 non-diarrheal stool specimens diluted in 1:5 in sterile phosphate buffer, before and after lyophilization (Ct value)

**Sample n°**	**1**	**2**	**3**	**4**	**5**	**6**	**7**	**8**	**9**	**10**	**11**	**12**	**13**	**14**	**15**
Without lyophilization	33.01	22.22	27.99	NA	NA	22.76	42.97	31.76	27.71	27.82	28.18	38.91	35.02	32.25	30.5
With lyophilization	15.61	24.24	21.70	17.41	25.19	16.78	22.02	25.94	20.58	21.47	21.24	27.43	37.34	33.91	24.28

NA, not amplified.

In a second step, the 41 diarrheal stool specimens were treated as described above: stools specimens were divided into two aliquots, one aliquot was lyophilized before DNA extraction and the second aliquot was not lyophilized. Lyophilization, DNA extraction and real-time PCR protocols were performed as described above. *M. smithii* was detected in 26/41 (63.4%) non-lyophilized versus 39/41 (95.1%) lyophilized aliquots (Ct value 22.04 ± 5.5) (Table 4); bacterial 16S rRNA gene was detected in 33/41 (80.5%) non-lyophilized aliquots (Ct value 28.11 ± 5.9) versus 40/41 (97.6%) lyophilized aliquots (Ct value 24.94 ± 6.6) (Table 5); and *S. enterica*-DNA detection was negative in 50/50 (100%) culture-negative specimens and was detected in 6/6 (100%) non-lyophilized and lyophilized aliquots (Ct value of 26.98 ± 4.55 and 26.16 ± 4.97, respectively) (Table 6). The proportion of positive specimens was significantly higher after lyophilization for the detection of *M. smithii* (p = 0.00043) and Bacteria (p = 0.015) but not for *S. enterica*. For positive specimens, this protocol increased Ct values of 6.9 Ct for the detection of *M. smithii,* 5.2 Ct for the detection of Bacteria and 0.8 Ct value for the specific detection of *S. enterica.*

**Table 4:** ***M. smithii *real-time PCR detection in 41 diarrheal stool specimens, before and after lyophilization (Ct value)**

**Sample n°**	**16**	**17**	**18**	**19**	**20**	**21**	**22**	**23**	**24**	**25**	**26**	**27**	**28**	**29**	**30**
Without lyophilization	NA	25.55	NA	18.87	39.33	36.94	37.84	37.69	31.95	38.51	39.22	40.32	24.18	36.14	NA
With lyophilization	38.98	18.82	35.20	16.29	25.62	23.21	26.54	28.51	24.41	23.96	25.2	23.67	23.65	23.98	17.5
**Sample n°**	**31**	**32**	**33**	**34**	**35**	**36**	**37**	**38**	**39**	**40**	**41**	**42**	**43**	**44**	**45**
Without lyophilization	34.83	NA	NA	33.91	NA	36.45	18.55	NA	37.05	22.02	39.05	19.28	33.16	19.25	NA
With lyophilization	21.53	22.47	19.34	27.19	18.30	27.48	21.69	24.49	24.14	20.53	23.88	19.18	13.92	19.27	24.44
**Sample n°**	**46**	**47**	**48**	**49**	**50**	**51**	**52**	**53**	**54**	**55**	**56**				
Without lyophilization	NA	NA	35.67	22.78	NA	19.2	15.97	19.59	13.92	17.98	18.4				
With lyophilization	18.08	29.99	NA	14.52	12.63	19.23	15.32	18.52	13.57	18.25	18.27				

**Table 5:** All bacteria real-time PCR detection with a universal system in 41 diarrheal stool specimens, before and after lyophilization (Ct value)

**Sample n°**	**16**	**17**	**18**	**19**	**20**	**21**	**22**	**23**	**24**	**25**	**26**	**27**	**28**	**29**	**30**
Without lyophilization	NA	NA	30.54	NA	28.83	34.47	35.95	36.64	27.85	28.04	34.85	34.02	NA	30.14	26.82
With lyophilization	34.65	17.44	26.86	29.49	28.21	24.99	23.43	39.07	22.81	25.02	22.53	32.33	25.78	28.33	26.36
**Sample n°**	**31**	**32**	**33**	**34**	**35**	**36**	**37**	**38**	**39**	**40**	**41**	**42**	**43**	**44**	**45**
Without lyophilization	33.42	26.45	34.22	NA	34.63	30.36	28.49	26.98	33.14	23.75	NA	22.79	31.95	31.05	33.50
With lyophilization	21.18	22.03	21.79	26.01	21.84	24.4	29.74	23.24	28.85	21.29	33.43	19.31	21.41	32.68	22.23
**Sample n°**	**46**	**47**	**48**	**49**	**50**	**51**	**52**	**53**	**54**	**55**	**56**				
Without lyophilization	29.24	NA	NA	24	23.21	23.05	17.59	19.94	14.65	18.33	18.58				
With lyophilization	20.62	35.95	43.68	22.91	10.82	23.24	16.33	19.66	14.43	19.15	19				

NA, not amplified. The proportion of positive specimens was significantly higher after lyophilization for the detection of **Bacteria** (p = 0.015).

**Table 6:** **Positive real-time PCR detection of *S. enterica *in 6 diarrheal stool specimens, before and after lyophilization (Ct value)**

**Sample n°**	**51**	**52**	**53**	**54**	**55**	**56**
Without lyophilization	31.65	28.14	26.35	18.39	29.23	28.09
With lyophilization	31.75	26.93	26.52	16.85	26.59	28.34

There was no statistically significant difference.

Our results were validated by the fact that all of the negative controls remained negative in all of the experiments. Also, reproducible values were obtained in duplicate. Dehydratation of stool has been shown to prevent DNA hydrolysis on human non-diarrheic fecal samples [15]. Previous studies also reported that lyophilization of pig and bovine stool specimens significantly improved the sensitivity of enteropathogenic bacteria detection with a 1.5- to 2-fold increase in DNA recovery compared to fresh stool specimens [16,17]. Data presented here showed that in human also, the lyophilization of stool specimens prior to DNA extraction increased the sensitivity of real-time PCR-based detection of archaeal and bacterial DNA. The effect of this protocol on the immunoassays has not been tested here; indeed, immunoassays are intended to provide a rapid result in a point-of-care situation [18], whereas the protocol here reported takes 48 hours to be completed.

Interestingly, the favorable effect of the lyophilization was more important for diarrheal stool specimens than for non-diarrheal specimens. Lyophilization could be especially useful for the molecular detection of enteropathogens which are in low-abundance in human diarrheal stool specimens such as *Salmonella* which is present at 10^3^ organisms/mL [19] and *Shigella* and *Vibrio cholerae* which are present at 10^1^-10^2^ organisms/mL [20,21]. In this work we targeted *M. smithii* and Bacteria that are present in high abundance but also *S. enterica* that is a low- abundance pathogen. Although the impact of the protocol reported here was not evaluated on viruses, viral pathogens are in great abundance in diarrheic stools and do not pose real problem for their PCR-based detection.

Previous studies showed the importance of using the acid-washed beads to lyse organisms with a thick cell wall such as *M. smithii*[22] and *Mycobacterium tuberculosis*[23]. The protocol reported here also incorporated mechanical lysis prior to DNA extraction; accordingly, *M. smithii* was detected in 93.3-95.1% specimens, a value consistent with the reported 95.7% prevalence of *M. smithii* in the general population in France [22].

The protocol here reported, combining lyophilization and a semi-automated DNA extraction, could be used for the routine detection of enteropathogen DNA in diarrheal stool specimens and the molecular diagnosis of infectious diarrhea [24].

## Competing interests

The authors declare that they have no competing interests.

## Authors’ contributions

ED performed analyses; ED and MD interpreted data and wrote the draft. Both authors read and approved the final manuscript.
